# ID 311 - Elaboração de um Guia para Padronização da Remume: a experiência do município de Fortaleza, Ceará

**DOI:** 10.5327/2237-9622.2025.v34s1.311

**Published:** 2025-11-25

**Authors:** Nívia Tavares Pessoa de Souza, Ana Cláudia Brito Passos, Mirian Parente Monteiro, Vitória Janaina Sousa de Assis Nunes, Ana Luíza Ribeiro Aguiar, Ana Michellane Gomes Costa do Nascimento, Antonia Jaqueline Nobre Bezerra, Diego Pinheiro Mesquita, Elise Feitosa Aragão, Francisco de Oliveira Silva, Isac Santiago de Araújo, Joyce de Pontes Gomes, Kelly Sisiane de Moraes Menezes, Letícia Karen Correia de Almeida, Mônica Helena Santos Sousa, Mônica Maria Monteiro Mesquita, Raimundo Jeová Martins Mesquita, Elisiane Freitas Bandeira, Mikaele Castro Sipriano, Giovana Karen Lira Nascimento

**Keywords:** seleção de medicamentos, Atenção Primária à Saúde, Avaliação de Tecnologias em Saúde, Comissão de Farmácia e Terapêutica

## Abstract

**Introdução:**

O estabelecimento de uma Relação Municipal de Medicamentos (Remume) implica a seleção de medicamentos que atendam às necessidades de saúde de um território. Dispor de métodos padronizados para atualização da Remume pode auxiliar na definição de critérios técnicos bem definidos e reprodutíveis, garantindo a eficácia e a segurança dos medicamentos selecionados. Este trabalho objetiva descrever o processo de elaboração de um guia para padronização do método de atualização da Remume do município de Fortaleza, Ceará.

**Método:**

A Comissão de Farmácia e Terapêutica (CFT) adaptou o método proposto pelo Ministério da Saúde (MS), com algumas alterações considerando a realidade do município. A padronização do método de análise considerou critérios relacionados à essencialidade dos fármacos, o perfil de atendimentos às condições de saúde, a experiência do município de Fortaleza na Atenção Primária à Saúde (APS) e o fortalecimento da Rede de Atenção à Saúde (RAS.) As etapas da metodologia foram: i) identificação dos medicamentos para análise de inclusão ou exclusão; ii) definição de prioridade dos medicamentos para elaboração de pareceres técnicos (PTs); iii) elaboração dos PTs dos medicamentos priorizados; e iv) submissão dos PTs para a apreciação da CFT. As etapas foram executadas pelos farmacêuticos da Coordenadoria de Assistência Farmacêutica (Coaf) com apoio técnico do Grupo de Prevenção ao Uso Indevido de Medicamentos da Universidade Federal do Ceará (Gpuim/UFC).

**Resultados:**

A elaboração do guia ocorreu concomitantemente ao processo de atualização da Remume. Na análise de inclusão/exclusão, consideraram-se: demandas internas das áreas técnicas da Secretaria Municipal de Saúde (SMS); demandas dos profissionais da RAS; itens não padronizados mais solicitados por processos administrativos; itens não padronizados mais prescritos na APS e medicamentos do Grupo 3 do Componente Especializado da Assistência Farmacêutica (Ceaf) não contemplados na Remume, conforme protocolos atendidos no município. Para a priorização, utilizou-se um fluxograma de decisão que considerou: presença do medicamento na Relação Nacional de Medicamentos Essenciais (Rename); presença do medicamento no Componente Básico da Assistência Farmacêutica (Cbaf), e se o medicamento possuía perfil de utilização na APS. Aplicaram-se critérios de essencialidade para os medicamentos priorizados por meio de um fluxograma de decisão. Para os medicamentos classificados como essenciais, elaborou-se PT utilizando os critérios: carga de doença, disponibilidade de alternativas, benefício potencial da tecnologia, perfil de segurança da tecnologia e consequências econômicas. Para a definição dos pesos desses critérios, a CFT utilizou o método de Lawshe.

**Conclusão:**

Todas as etapas foram testadas em conformidade com as adaptações ao método proposto pelo MS. A CFT definiu a pontuação dos atributos dos critérios utilizados. Para o critério "carga da doença", utilizaram-se os dados do município/estado e, na ausência desses, os dados do País. A CFT propôs para as consequências econômicas a variação de uma a três vezes do valor per capita habitante/ano gasto no município, relativo ao ano de 2023, e definindo sete faixas de valores. Para validar a pertinência dessas faixas, utilizou-se o custo/tratamento dos medicamentos presentes na Remume 2022. O método proposto mostrou-se válido e reprodutível e garantirá um padrão de qualidade para as próximas revisões da Remume.

